# Manipulation of *in vivo* iron levels can alter resistance to oxidative stress without affecting ageing in the nematode *C. elegans*

**DOI:** 10.1016/j.mad.2012.03.003

**Published:** 2012-05

**Authors:** Sara Valentini, Filipe Cabreiro, Daniel Ackerman, Muhammed M. Alam, Micha B.A. Kunze, Christopher W.M. Kay, David Gems

**Affiliations:** a Institute of Healthy Ageing, and Research Department of Genetics, Evolution and Environment, University College London, Gower Street, London WC1E 6BT, UK; b Institute of Structural and Molecular Biology, University College London, Gower Street, London WC1E 6BT, UK; c London Centre for Nanotechnology, University College London, 17-19 Gordon Street, London WC1H 0AH,UK

**Keywords:** *C. elegans*, Iron homeostasis, Fenton reaction, Oxidative damage, Ageing, EPR spectroscopy

## Abstract

Iron-catalyzed generation of free radicals leads to molecular damage *in vivo*, and has been proposed to contribute to organismal ageing. Here we investigate the role of free iron in ageing in the nematode *Caenorhabditis elegans*. Media supplementation with Fe(III) increased free iron levels *in vivo*, as detected by continuous-wave electron paramagnetic resonance spectroscopy and elevated expression of the iron-sensitive reporter transgene *pftn-1::gfp*. Increased free iron levels caused elevated levels of protein oxidation and hypersensitivity to *tert*-butyl hydroperoxide (*t*-BOOH) given 9 mM Fe(III) or greater, but 15 mM Fe(III) or greater was required to reduce lifespan. Treatment with either an iron chelator (deferoxamine) or over-expression of *ftn-1*, encoding the iron sequestering protein ferritin, increased resistance to *t*-BOOH and, in the latter case, reduced protein oxidation, but did not increase lifespan. Expression of *ftn-1* is greatly increased in long-lived *daf-2* insulin/IGF-1 receptor mutants. In this context, deletion of *ftn-1* decreased *t*-BOOH resistance, but enhanced both *daf-2* mutant longevity and constitutive dauer larva formation, suggesting an effect of ferritin on signaling. These results show that high levels of iron can increase molecular damage and reduce lifespan, but overall suggest that iron levels within the normal physiological range do not promote ageing in *C. elegans*.

## Highlights

► Here we test whether iron-catalyzed oxidative damage contributes to organismal ageing. ► We develop new methodologies to measure free iron *in vivo* in *C. elegans*. ► Moderate iron supplementation can increase oxidative damage without reducing lifespan. ► Iron chelation or increasing ferritin levels increase resistance to oxidative stress but do not increase lifespan. ► Our findings argue against the oxidative damage theory of ageing.

## 1 Introduction

### 1.1 The oxidative damage theory of ageing

The mechanisms underlying the ageing process remain poorly defined, although many theories have been proposed (Medvedev, 1990). Prominent among these is the idea that ageing results from the accumulation of damage to biomolecules caused by reactive oxygen species (ROS) (Harman, 1956; Sohal and Weindruch, 1996). Thus, far, extensive experimental probing of this using animal models has not, on the whole, resulted in its clear validation (Muller et al., 2007; Perez et al., 2009).

A number of tests of the oxidative damage theory have used the short-lived nematode *Caenorhabditis elegans* (Van Raamsdonk and Hekimi, 2010). In this organism in particular, the results of many such tests have not supported the theory. For instance, the gene *sod-2* encodes the major mitochondrial superoxide dismutase (SOD) (Doonan et al., 2008), an important antioxidant enzyme that removes the superoxide free radical (O_2_•^−^). Deletion of *sod-2* might be expected to increase oxidative damage levels and decrease lifespan. It does indeed increase oxidative damage but does not decrease lifespan, and can even increase it (Van Raamsdonk and Hekimi, 2009). In the same vein, treatment with compounds with SOD activity can increase resistance to oxidative stress, but does not increase *C. elegans* lifespan (Keaney and Gems, 2003; Keaney et al., 2004; Kim et al., 2008; Uchiyama et al., 2005), though it should be noted that one initial study did report an increase in lifespan (Melov et al., 2000). Furthermore, treatments that increase ROS production can increase rather than decrease lifespan (Heidler et al., 2010; Schulz et al., 2007; Yang and Hekimi, 2010). Taken together, these studies raise doubts about whether oxidative damage contributes to age-associated increases in pathology and mortality in *C. elegans* (Gems and Doonan, 2009).

### 1.2 Iron as a generator of oxidative damage

Iron plays a central role in many essential cellular processes including oxygen transport, xenobiotic detoxification, and mitochondrial energy metabolism. Iron is present *in vivo* in both the oxidized ferric(III) and reduced ferrous(II) forms. All cells contain a pool of “free” (uncomplexed) iron, which becomes toxic at high concentrations, particularly due to its ability to generate oxidative stress *via* the Fenton reaction. Here Fe(II) is oxidized by H_2_O_2_ to Fe(III) generating the highly reactive hydroxyl radical OH• (Gutteridge and Halliwell, 2000; Halliwell and Gutteridge, 1984). The iron-catalyzed Fenton reaction is a major source of OH• in biological systems (Fridovich, 1978; Keyer et al., 1995; Liochev, 1999; Meneghini, 1997), though other transition metals (*e.g.* copper) can also catalyze this reaction. Moreover, oxidative stress can disrupt iron homeostasis. For example, O_2_•^−^ can cause release of iron from Fe-S proteins, catalyzing increases in OH• levels *via* the Fenton reaction, further increasing ROS levels (Meneghini, 1997; Puntarulo and Cederbaum, 1996).

The oxidative damage theory predicts that free iron levels and iron homeostasis more broadly are likely to influence ageing rate (Galaris et al., 2008; Mwebi, 2005; Terman et al., 2006). More specifically, it suggests that elevated levels of free iron will increase the rate of ageing due to increased ROS production, while robust control of iron homeostasis will protect against it. It has therefore been speculated that iron chelation treatment might be protective against ageing (Polla et al., 2003; Polla, 1999). It is indeed the case that lowering levels of free iron can result in resistance to oxidative stress. For example, iron chelation results in resistance to H_2_O_2_ toxicity in *Escherichia coli* strains with mutations affecting SOD and oxidative damage repair enzymes (Imlay et al., 1988; Keyer et al., 1995).

### 1.3 The iron storage protein ferritin protects against oxidative stress

Ferritins are iron storage proteins that sequester large amounts of Fe(II), rendering it unavailable for Fenton chemistry, and as such are effective antioxidants (Levi and Arosio, 2004; Vile and Tyrrell, 1993). In vertebrates, ferritins assemble into 24 subunit protein nanospheres, each of which can store up to 4500 iron atoms in its central cavity (Crichton and Ward, 1992). Within this cavity, Fe(II) is oxidized to Fe(III) by the ferroxidase activity of heavy chain (H) ferritin. Here, iron interacts with oxygen, is oxidized to Fe(III) and then migrates to the cavity where it nucleates and aggregates to form the iron core.

*C. elegans* has two ferritins, FTN-1 and FTN-2 that contain predicted ferroxidase active sites. Expression of *ftn-1* and, to a lesser degree, *ftn-2* is induced by iron, and mutation of *ftn-1* results in hypersensitivity to iron toxicity, consistent with the role of ferritins in iron sequestration (Gourley et al., 2003; Kim et al., 2004). The main site of *ftn-1* expression is the intestine, while *ftn-2* is expressed in the body wall muscle, hypodermis and pharynx (but not the intestine) (Kim et al., 2004).

### 1.4 ftn-1 is up-regulated in long-lived daf-2 insulin/IGF-1 receptor mutants

Mutations of the *C. elegans* gene *daf-2*, which encodes an insulin/IGF-1 receptor, result in many phenotypic changes including extended lifespan (Kenyon et al., 1993; Kimura et al., 1997). How reduced insulin/IGF-1 signaling causes extended lifespan remains unclear. A potential clue is that mutations that reduce insulin/IGF-1 signaling (IIS) cause increased resistance to a range of stressors, including heat (Lithgow et al., 1995), pro-oxidant chemicals (Honda and Honda, 1999; Larsen, 1993; Vanfleteren, 1993) and ultraviolet radiation (Murakami and Johnson, 1996). This likely reflects up-regulation of various stress response genes (Lithgow and Walker, 2002). *daf-2* mutants also show a strong increase in *ftn-1* expression (Ackerman and Gems, 2012; McElwee et al., 2007). One possibility is that the resulting antioxidant effect contributes to *daf-2* longevity.

In this study, we explore whether iron homeostasis and free iron levels influence resistance to oxidative stress and ageing in *C. elegans*.

## 2 Materials and methods

### 2.1 Strains and culture conditions

The strains used in this study included the following: N2 (wild type), DR1567 *daf-2(m577), GA303 rrf-3(pk1426) daf-2(m577)*, GA631 *lin-15(n765ts) wuIs177 [Pftn-1::GFP, lin-15(+)]*, GA901 *(wuEx188 [coel::GFP])*, GA904 *(wuEx187 [Pftn-1::ftn-1::ftn-1 3′UTR + coel::GFP])*, GA912 *ftn-1(ok3625)* [6× out-crossed derivative of RB2603 *ftn-1(ok3625)*], GA931 *daf-2(m577); ftn-1(ok3625)*, GA933 *ftn-2(ok404); daf-2(m577); ftn-1(ok3625)*, NL2099 *rrf-3(pk1426)* and SS104 *glp-4(bn2)*. Nematodes were raised at 20 °C in 60 mm Petri dishes on nematode growth media (NGM), with *E. coli* OP50 bacteria as food source. To subculture lines with transgene arrays that were not chromosomally integrated (*e.g.* GA904 and GA901) worms were picked that carried the *coel*::GFP marker.

### 2.2 Transgenic line construction

To obtain transgenic *C. elegans* containing multiple copies of the *ftn-1* gene, a 5990 bp PCR product containing the *ftn-1* gene (including 3860 bp upstream and 1048 bp downstream of the coding region) was injected into wild-type worms (Evans, 2006). This was co-transformed with the marker construct *coel::GFP*, which causes bright green fluorescence in the coelomocytes (Miyabayashi et al., 1999), macrophage-like cells found within the nematode pseudocoelom. The *ftn-1* gene was amplified from *C. elegans* gDNA template using the following primers. Forward primer: ftn-1.5in TGTAGGGTTTGATTGTGGTTTG, reverse primer: ftn-1.4in_rev2 AAATTCGGAAATGTCGCAGC.

### 2.3 Ferric ammonium citrate (FAC) and deferoxamine (DF) supplementation

All iron (FAC) and iron chelator (DF) treatments were performed on NGM plates supplemented with FAC (C_6_H_8_O_7_·nFe·nH_3_N) and the iron chelator DF (Desferal)(C_25_H_48_N_6_O_8_). The appropriate amount of FAC (5–50 mM) and DF (100 μM) was added to the molten NGM agar just prior to pouring the plates.

### 2.4 GFP measurements

To quantify the GFP expression in the *Pftn-1::GFP* reporter strain, young adult worms were picked in 96 well V-shaped microtitre plates (Greiner) and GFP was measured in a GENios Plus microplate reader (Tecan) at excitation and emission wavelengths of 395 nm and 535 nm, respectively.

### 2.5 EPR sample preparation and measurement

To obtain a measure of free iron *in vivo* in *C. elegans*, we used continuous-wave electron paramagnetic resonance (cw-EPR) spectroscopy to detect Fe(III). EPR detects and fingerprints molecular species containing unpaired electrons using a combination of a fixed frequency microwave radiation, *ν*, and an applied magnetic field, *B*_0_. Transitions appear at magnetic field positions determined by the equation: *hν* = *gμ*_0_*B*_0_, where *h* is Planck's constant, *μ*_0_ is the Bohr magneton, and *g* is the g value or more properly, the *g* tensor. This has three principle components, the values of which depend on the precise electronic structure and thus are unique for every system. When all 5 *d* electrons in Fe(III) are in different orbitals, known as a high-spin state, one of these components has a value around 4.3 and is readily observed in the spectrum. EPR has previously been used to detect increased free iron levels in SOD knockout strains of *E. coli* and yeast (Keyer and Imlay, 1996; Srinivasan et al., 2000), and has been used to estimate free iron levels in *C. elegans* (Pate et al., 2006).

*C. elegans* whole worm samples were prepared for EPR as previously described (Pate et al., 2006). Synchronized populations of young adult *C. elegans* were raised on agar plates, and then collected in M9 buffer, washed several times and centrifuged for 3 min at 4000 rpm at 4 °C. The supernatant was removed and the pellet of worms resuspended in 15% glycerol. Deferoxamine was added to a final concentration of 2 mM, and worms were then incubated for 15 min at room temperature. During this incubation the samples were transferred to EPR tubes using 310 mm glass Pasteur pipettes. EPR tubes were then placed on ice to hasten the accumulation of worms at the bottom of the tube. The supernatant was then removed and the sample flash frozen and stored in liquid nitrogen until use.

We introduced a new step of normalising the EPR spectra to the manganese signal of the worms (Fig. S1). This internal control represented a more reliable way to compare the individual samples with each other than the counting method previously used (Pate et al., 2006). EPR measurements were performed on a Bruker EMXplus spectrometer operating at 9.4 GHz (*X*-band) equipped with a 4122SHQE resonator, with an Oxford Instruments ESR900 cryostat for measurements at 10 K. Measurements were performed with a magnetic field sweep from 0 to 6000 Gauss, a microwave power of 2 mW, a modulation amplitude of 10 Gauss and a modulation frequency of 100 kHz. The high spin rhombic ferric iron peak was detected at *g* = 4.3. Ferrous iron signals are too broad to be detected using EPR, so worms were treated with DF, which converts Fe(II) to Fe(III). Thus, the relative magnitude of the peak at *g* = 4.3 is a measure of the relative levels of both free Fe(II) and Fe(III). The previous study showed that the free iron pool in *C. elegans* consists of 50% Fe(II) and 50% Fe(III).

### 2.6 Carbonylated protein detection

Protein oxidation (carbonylation) levels of young adult worms were measured much as previously described (Yang et al., 2007) using an Oxyblot assay kit (Millipore) according to manufacturer's protocol. 1-day-old worms were collected and homogenized using a Bioruptor (Cosmo Bio Co., Ltd., Tokyo, Japan) in 2-ml microcentrifuge tubes containing equal amounts of suspended worms and CelLytic (Sigma) and 1× protease inhibitors (Roche). The resulting homogenate was spun at 20,000 rpm for 30 min at 4 °C, and the supernatant collected. Samples (15 μg) of total protein extract were incubated for 15 min at room temperature with 2,4-dinitrophenylhydrazine to form the carbonyl derivative dinitrophenylhydrazone before SDS–PAGE separation. After transfer onto nitrocellulose, carbonylated proteins were detected by anti-dinitrophenol antibodies. Blots were developed using the SuperSignal West Pico chemiluminescent substrate (Perbio Sciences). Films were scanned and the density of each band or the entire lane was quantified by densitometry using ImageQuant TL (GE Healthcare Europe GmbH).

### 2.7 Lifespan measurements

Age-synchronous worms (hermaphrodites) were transferred to test plates (NGM alone, or supplemented with iron or iron chelator, or RNAi plates) and scored every second day. /glp-4(bn2)/ or /rrf-3(pk1426)/ worms were transferred to fresh plates every day during the first week. In some trials, 5-fluoro-2′-deoxyuridine (FUdR) was used to prevent progeny production. In these cases, FUdR was applied to the surface of plates with *E. coli* lawns already grown, to a final concentration of 10 μM one day prior to the start of the lifespan experiment. The L4 stage was used as *t* = 0 for lifespan analysis. When a worm failed to move after touching, it was removed from the plate and scored as dead. Loss where animals crawled off the plate, bagged (*i.e.* internal hatching of embryos) or showed uterine rupture were treated as censored values.

### 2.8 Oxidative stress assays

Worms were transferred as late L4s or young adults to NGM plates containing 10 mM *tert*-butyl hydroperoxide (*t*-BOOH) and scored for survival every 1–2 h (Tullet et al., 2008).

### 2.9 RNA-mediated interference (RNAi)

RNAi by feeding was performed as previously described (Kamath et al., 2001). RNAi *E. coli* feeding clones were derived from the Ahringer Library (Kamath et al., 2003; Kamath and Ahringer, 2003) kindly provided by Dr. Steven Nurrish. Worms were maintained for two generations on the RNAi feeding clones prior to testing. For simultaneous RNAi of *ftn-1* and *ftn-2*, agar plates were spread with 50:50 mixtures of *E. coli* HT115 bearing the respective feeding plasmids.

### 2.10 Quantitative real-time RT-PCR

RNA was isolated from synchronized worms in the L4/young adult stage using TRIzol (Invitrogen). 1 μg of RNA was transformed into cDNA using the SuperScript™ II RNase H reverse transcriptase kit (Invitrogen). 1 μl of this cDNA was used as template for RT-PCR using ABI SYBR Green PCR mix (Applied Biosciences) and an ABI 7000 RT-PCR machine.

### 2.11 Statistics

Statistical comparisons between survival of nematodes under different treatments were performed with the JMP 7.0.1 programme (SAS Institute Inc.) using the log rank test. For analysis of the GFP-expression measurements, protein carbonyl measurements, brood size and EPR measurements, the Student's *T* test (two-tailed) was used.

## 3 Results

### 3.1 Moderate increases in ‘free’ iron can cause oxidative stress without reducing lifespan

To test the effect of increased free iron levels in *C. elegans*, we supplemented their media with iron (ferric ammonium citrate, FAC). We first tested effects of additional iron on relative levels of free iron within the worms using cw-EPR spectroscopy (Fig. S1). The results implied that supplementation with FAC to 15 mM increased *in vivo* free iron levels by 328% (*p* = 0.0004) (Fig. S2A and B). Individual measurements of worms exposed to 5 mM and 50 mM FAC showed increases in free iron of 34% and 540%, respectively (Fig. S2A and B, Table S1).

It was previously shown that iron supplementation increases *ftn-1* expression, measured as mRNA levels (Gourley et al., 2003). We tested whether increased iron led to elevated expression of a *Pftn-1::GFP* transcriptional reporter, in *C. elegans* strain GA631 (Ackerman and Gems, 2012). Exposure to 25 mM FAC increased *Pftn-1::GFP* fluorescence by 146% (*p* = 0.0002). Thus, all other things being equal, *Pftn-1::GFP* expression can provide an indication of free iron levels *in vivo*.

Increased free iron levels are predicted to increase levels of hydroxyl radicals due to Fenton chemistry. Consistent with this, iron supplementation from L4 stage caused reduced resistance to peroxide stress (*tert*-butyl hydroperoxide, *t*-BOOH) in 1-day-old adults, suggesting increased production of *tert*-butyl hydroperoxyl radicals. Addition of 15 mM FAC caused a decrease in mean survival time of 16.4% (*p* < 0.0001) in wild-type worms on 10 mM *t*-BOOH (Fig. S2E, Table S2). Next we tested whether 15 mM iron lead to an increase in protein oxidation in 1-day-old adults. We found that it caused a 99.5% increase (*p* = 0.02) in levels of protein carbonylation (Fig. S2C and D).

Our results suggest that supplementation with 15 mM FAC increased levels of free iron *in vivo* which increases levels of ROS and of protein oxidation in *C. elegans*. To test whether this could affect ageing, we exposed worms to 15 mM FAC throughout their life. This resulted in a 27.7% decrease (*p* < 0.0001) in lifespan relative to non-supplemented controls (Fig. S2F, Table S3).

**Fig. 1:**
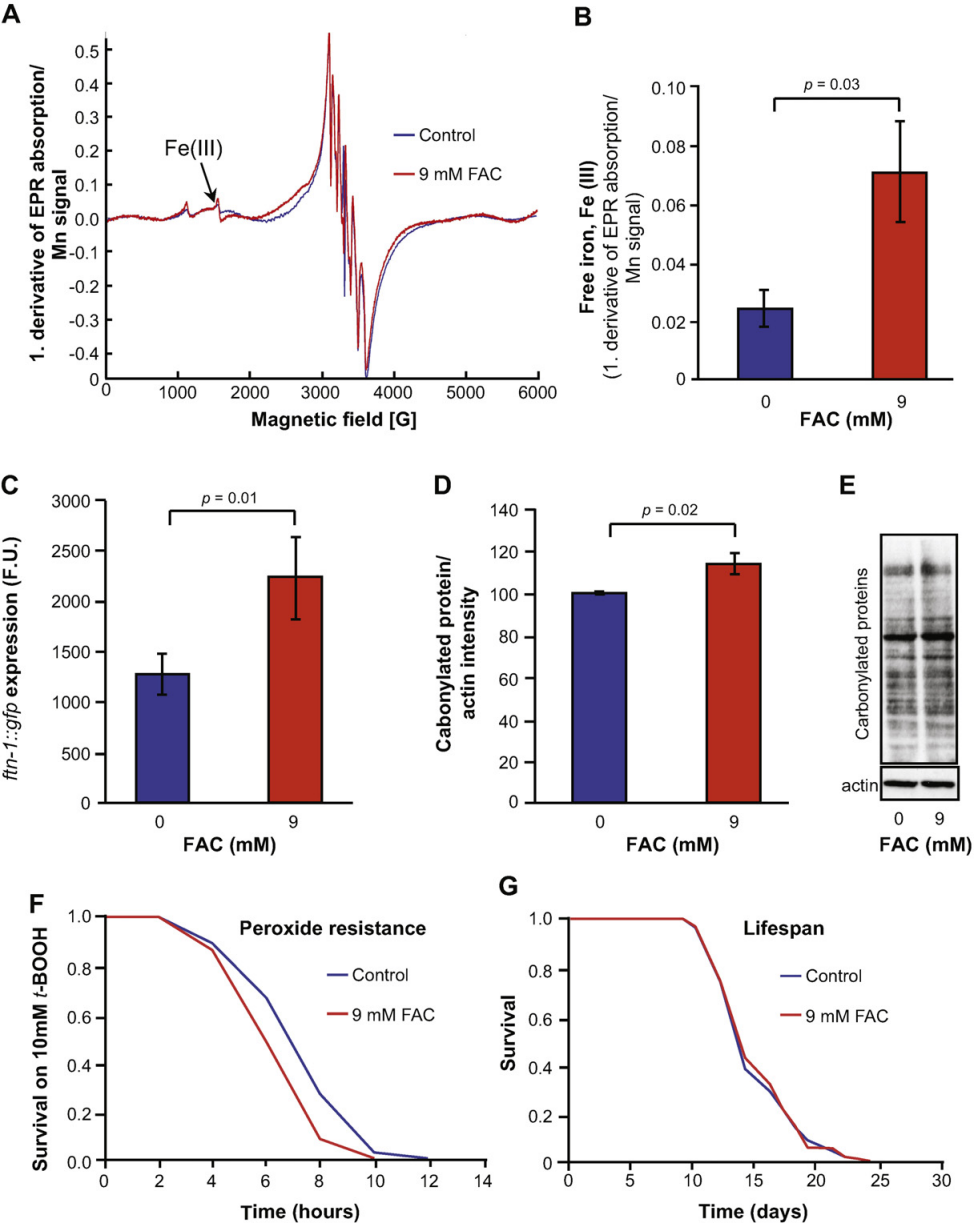
Effect of moderate iron supplementation (9 mM FAC) on *C. elegans*. (A and B) 9 mM ferric ammonium citrate (FAC) significantly increases free iron levels *in vivo*, shown using cw-EPR spectroscopy. (C) *Pftn-1::GFP* expression is increased by exposure to 9 mM or higher concentrations of FAC; F.U., fluorescence units. (D and E) Protein oxidation is increased by iron supplementation. (D) Statistical analysis of three biological replicates showing carbonylated protein levels normalized to actin immunoreactivity. Error bars, S.E.M. (E) Representative oxyblot. With 9 mM FAC, *t*-BOOH toxicity is increased (F), but lifespan is not reduced (G).

The shortening of lifespan by 15 mM FAC could reflect either an acceleration of the ageing process, or the action of a mechanism unrelated to normal ageing. To probe this, we asked whether there exist levels of iron supplementation that increase peroxide sensitivity and levels of protein oxidation without shortening lifespan (Tables S1–S5). We found that given administration of 9 mM FAC to *C. elegans*, EPR detected an increase in free iron levels *in vivo* of 192.8% (*p* = 0.03) (Fig. 1A and B, Table S1). 9 mM FAC increased *Pftn-1::gfp* expression by 75.3% (*p* = 0.01, Fig. 1C) and also caused a decrease in *t*-BOOH resistance (Fig. 1F, Table S4) and an 18% increase in protein oxidation (*p* = 0.02) in one day old adults (Fig. 1D and E), implying that it increases ROS production. However, 9 mM FAC did not shorten lifespan (Fig. 1G, Table S5). This suggests that under standard, non-FAC supplemented culture conditions, free iron levels do not contribute to ageing.

### 3.2 Reducing iron levels increases oxidative stress resistance but not lifespan

**Fig. 2:**
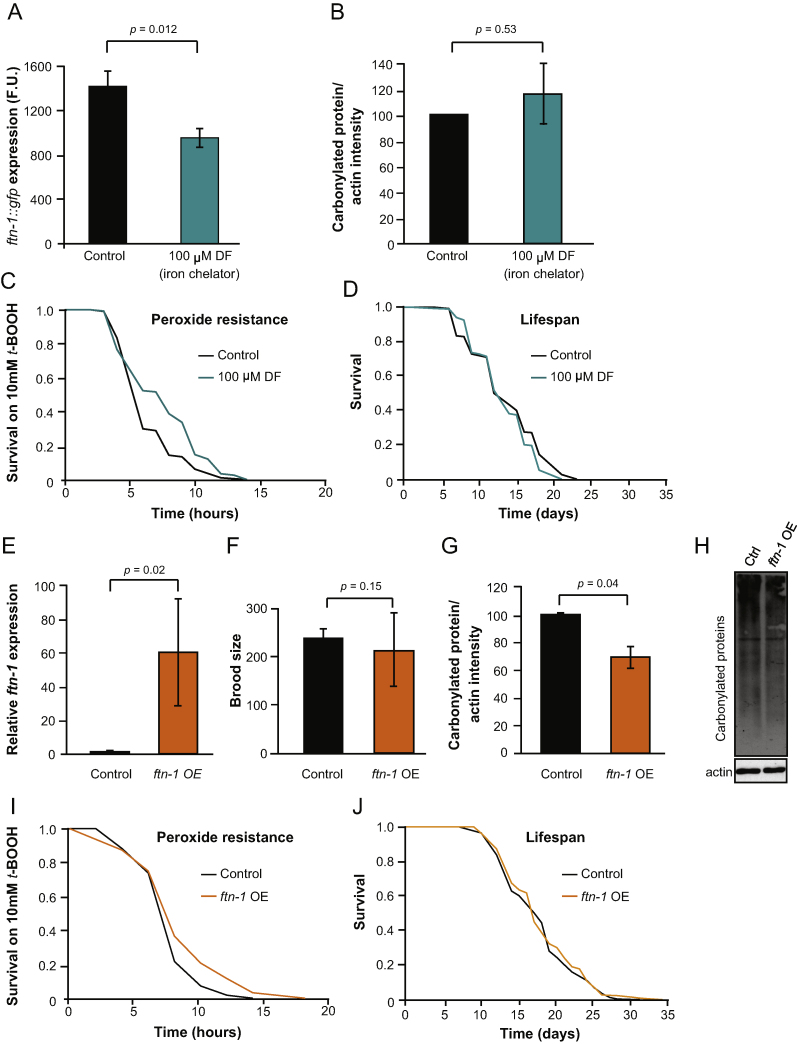
Effects of lowering free iron levels in *C. elegans*. (A) Iron chelation (100 μM deferoxamine, DF) reduces *pftn-1::gfp* expression (F.U., fluorescence units). The columns represent the mean GFP measurements of three biological replicates. Error bars, S.E.M. (B) No effect of iron chelation on protein oxidation. Statistical analysis of four biological replicates showing carbonylated protein levels normalized to actin immunoreactivity. Error bars, S.E.M. (C) Iron chelation increased resistance to peroxide (*t*-BOOH), but (D) did not affect lifespan. (E) *wuEx187* increased *ftn-1* mRNA levels relative to *wuEx188* (marker control). (F) Over-expression (OE) of *ftn-1* did not affect fertility, but it significantly reduced protein oxidation levels (G, H). Error bars, S.E.M. (I) *ftn-1* OE increased resistance to peroxide, but (J) did not increase lifespan.

If free iron contributes to *C. elegans* ageing under standard culture conditions, then lowering iron levels ought to increase lifespan. To test this idea, we took two different approaches to reduce irons levels: administration of an iron chelator, and forced over-expression of *ftn-1*. The iron chelator deferoxamine (DF, 100 μM from late L4 stage onwards) reduced *Pftn-1::GFP* expression in 1-day-old adults by 32.7% (*p* = 0.01, Fig. 2A), suggesting that DF lowers *in vivo* free iron levels in *C. elegans*. EPR did not detect a change in free iron level after iron chelation (data not shown), but this may reflect a lack of sensitivity in the technique (see Section 4).

We then constructed a *C. elegans* strain, GA904, over-expressing *ftn-1* from an extra-chromosomal transgene array, *wuEx187 [Pftn-1::ftn-1::ftn-1 3*′*UTR + coel::GFP]* (see Section 2). RT-PCR confirmed that levels of *ftn-1* mRNA are greatly increased in this strain (Fig. 2E). GA904 appeared healthy, and showed a normal level of fertility (Fig. 2F) suggesting that elevated *ftn-1* expression has no major deleterious effects.

The transgene arrays *wuEx187* and *wuEx188*, which carries the injection marker *coel::GFP* alone, were crossed into a *Pftn-1::GFP* background to try to gauge whether free iron levels are reduced by *ftn-1* over-expression. However, *wuEx187* actually increased *Pftn-1::GFP* expression (data not shown), suggesting the presence of as yet uncharacterized complexity in the regulation of *ftn-1* expression.

However, both iron chelation and *ftn-1* over-expression resulted in increased peroxide resistance, consistent with reduced free iron levels *in vivo*. Iron chelation increased survival time on *t*-BOOH by 14% (*p* = 0.008) (Fig. 2C, Table S6) and *ftn-1* over-expression by 11% (*p* = 0.005) (Fig. 2I, Table S7). *ftn-1* over-expression (but not iron chelation) also reduced protein oxidation levels by 31% (*p* = 0.03) (Fig. 2B, G, and H). However, neither treatment produced any effect on lifespan (Fig. 2D, J; Table S8 and S9). These findings are consistent with the view that, under standard culture conditions, free iron levels affect oxidative stress resistance, but not ageing.

### 3.3 Effects of loss of ftn-1 on oxidative stress resistance and lifespan

*daf-2* mutant worms are resistant to oxidative stress (Oxr) (Honda and Honda, 1999) and long-lived (age) (Kenyon et al., 1993) and show elevated *ftn-1* expression (Ackerman and Gems, 2012; McElwee et al., 2004, 2007). They also exhibit, conditional, constitutive formation of diapausal dauer larvae (Daf-c), which are also stress resistant and long-lived (Hu, 2007). Moreover, dauer larvae show elevated *ftn-1* mRNA levels relative to recovering dauers (Wang and Kim, 2003). In principle, elevated *ftn-1* expression could contribute to *daf-2* Oxr and/or Age phenotypes. To explore this, we examined the effect of abrogating *ftn-1* function on *daf-2(m577)* and *daf-2*(+) worms. *ftn-1* gene function was blocked using either RNAi in combination with the RNAi-sensitising mutation *rrf-3(pk1426)*, or the *ftn-1(ok3625)* deletion mutation, which is nullimorphic [*ftn-1(0)*]. *ok3625* was back-crossed six times into N2 prior to testing to reduce effects of genetic background.

**Fig. 3:**
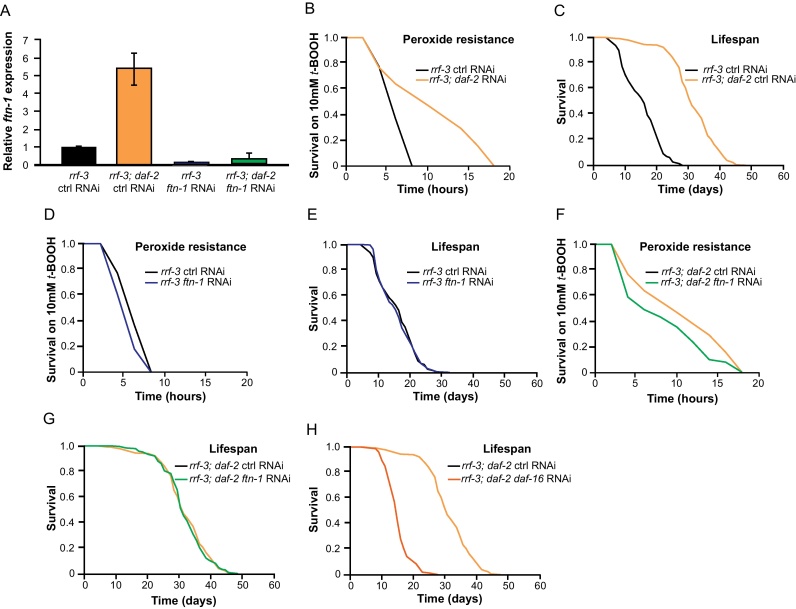
Role of ferritin in *daf-2* mutant phenotypes. (A) Effect of *ftn-1* RNAi on *ftn-1* mRNA levels in *rrf-3(pk1426)* and *rrf-3(pk1426); daf-2(m577)* strains. Means of two biological replicates, hence statistical comparisons could not be conducted. Error bars, S.E.M. (B) Increased peroxide resistance in *daf-2* mutant. (C) Increased lifespan in *rrf-3; daf-2* relative to *rrf-3*. (D-G) Effects of *ftn-1* RNAi on peroxide resistance and lifespan. (D) Peroxide resistance is reduced (*p* = 0.01, Table S13). (E) No effect on lifespan (*p* = 0.98, Table S12). (F) Peroxide resistance is reduced, although the reduction did not quite reach statistical significance (*p* = 0.06, Table S13). (G) No effect on lifespan (*p* = 0.72, Table S12). (H) RNAi of *daf-16* shortens lifespan of *rrf-3; daf-2* mutant (positive control).

We first verified that *daf-2(m577)* mutants show elevated *ftn-1* mRNA levels, and are Oxr (*t*-BOOH resistant) and Age (Fig. 3A–C). Efficacy of *ftn-1* RNAi was checked by RT-PCR (Fig. 3A). This indicated 96% and 95.5% reductions in *ftn-1* mRNA in *daf-2*(+) and *daf-2(m577)* worms, respectively. *ftn-1* RNAi caused a slight reduction in *daf-2 t*-BOOH resistance, although this did not quite reach statistical significance (*p* = 0.06, Fig. 3F, Table S11). In *daf-2*(+) worms, *ftn-1* RNAi clearly reduced *t*-BOOH resistance (11.1% reduction in mean survival time, *p* = 0.01, Fig. 3D). In neither *daf-2*(+) or *daf-2(m577)* worms did *ftn-1* RNAi affect lifespan (Fig. 3E and G, Table S10). By contrast, RNAi of *daf-16* (positive control) suppressed *daf-2* Age (Fig. 3K). These results suggest, again, that iron homeostasis is important for stress resistance but not lifespan.

However, tests with *ftn-1(0)* gave different results. *ftn-1(0)* markedly reduced *daf-2(m577)* Oxr but, surprisingly, did not affect *t*-BOOH resistance in *daf-2*(+) worms (Fig. S3D and F, Table S12). Also unexpectedly, *ftn-1(0)* enhanced *daf-2(m577)* Age, but decreased lifespan in *daf-2*(+) worms (Fig. S3C and E, Table S13).

Deletion of the *sod-4* extracellular superoxide dismutase (SOD) gene was previously shown to enhance both *daf-2(m577)* Age and Daf-c, suggesting that *sod-4* exerts redox effects on insulin/IGF-1 signaling (Doonan et al., 2008). One possibility is that the enhancement of *daf-2(m577)* Age by *ftn-1(0)* reflects a similar mechanism. Consistent with this, *ftn-1(0)* enhanced the Daf-c phenotype, increasing dauer formation at 23 °C from 6% to 23%, although this difference did not reach statistical significance, likely due to the typically high variation between individual Daf-c assays (*p* = 0.12) (Table S14).

One possibility is that there exists functional redundancy between *ftn-1* and the second ferritin gene *ftn-2*. While *ftn-1(0)* enhanced *daf-2(m577)* Age, *ftn-1(0)* and *ftn-2(0)* together showed no effect (Table S13). Moreover, in a *daf-2*(+) background, *ftn-2(0); ftn-1(0)* reduced lifespan by 16% more than *ftn-1(0)* alone (*p* < 0.0001). However, simultaneous RNAi of *ftn-1* and *ftn-2* did not reduce lifespan in *daf-2*(+) worms (data not shown).

The effects of loss of *ftn-1* on lifespan described here are hard to interpret, since depending on genetic background, and mode of abrogation, reduced *ftn-1* expression either increases, decreases or has no effect. However, our results suggest that deletion of *ftn-1* alters signaling in *daf-2* mutants, thereby enhancing Daf-c and Age. They also provide further evidence that *ftn-1* contributes to resistance to oxidative stress in a *daf-2* mutant background.

## 4 Discussion

### 4.1 Increased free iron can cause oxidative stress without reducing lifespan

In this study we have investigated the role of iron and iron-mediated oxidative stress on ageing in *C. elegans*. We show that high levels of iron cause peroxide sensitivity, oxidative damage and reduced lifespan. Thus, iron at high levels (15 mM FAC or greater) can reduce lifespan, perhaps by increasing oxidative damage. However, many compounds are toxic at high concentrations. The critical question here is: are free iron levels a determinant of ageing under standard culture conditions?

We first asked whether there exist concentrations of iron that increase peroxide sensitivity and oxidative damage without reducing lifespan, and found that this was the case for 9 mM FAC. Our results suggested that at 9 mM FAC, increased Fenton chemistry leads to increased ROS production, increased oxidative damage, but this is not sufficient to shorten lifespan. This in turn suggests that levels of Fenton chemistry at FAC concentrations of 9 mM or below (*i.e.* standard iron concentrations) do not contribute to ageing.

### 4.2 Iron chelation and ftn-1 over-expression increase resistance to oxidative stress but do not increase lifespan

Overall, our results imply that both iron chelation and *ftn-1* over-expression lead to a reduction in levels of free iron *in vivo*. Both treatments resulted in resistance to peroxide, and *ftn-1* over-expression reduced levels of protein oxidation, consistent with lower levels of Fenton chemistry *in vivo*. However, neither treatment resulted in an increase in lifespan. This suggests that under standard conditions, neither levels of free iron or protein oxidation are critical determinants of ageing. The usefulness of these conclusions depends in part on the degree to which the influence of free iron levels on ageing is similar between nematodes and mammals. However, our findings do not support the view that iron chelation is an effective intervention to slow animal ageing.

### 4.3 Effects of loss of function of ftn-1 on stress resistance and lifespan

Our studies of effects of iron supplementation and treatments aimed at reducing free iron levels both imply that under normal culture conditions free iron is an important determinant of oxidative stress resistance but not of ageing. To test this further, we examined the effect of loss of function of *ftn-1* on oxidative stress resistance and lifespan in *daf-2*(+) and *daf-2(m577)* mutants. Here, however, largely different results were obtained depending on whether *ftn-1* function was abrogated by RNAi or by mutation. In *daf-2*(+) worms, *ftn-1* RNAi reduced resistance to *t*-BOOH but had no effect on lifespan; by contrast, *ftn-1(0)* had no effect on resistance to *t*-BOOH and reduced lifespan. In *daf-2(m577)* worms, both *ftn-1* RNAi and *ftn-1(0)* caused some reduction in resistance to *t*-BOOH, but only *ftn-1(0)* had an effect on lifespan – and it increased rather than decreased it.

How could similar interventions produce such different results? RNAi greatly reduces *ftn-1* expression levels, while *ftn-1(0)* removes it entirely. RNAi appears to reduce *ftn-1* function sufficiently to increase free iron, and thereby reduce resistance to *t*-BOOH. Deletion of *ftn-1* may disrupt iron homeostasis to a greater extent, such that viability is reduced. The absence of an effect of *ftn-1(0)* could imply that the severity of the mutation induces compensatory changes in expression of other genes, such as *ftn-2*. It could also reflect effects of *ftn-1* RNAi on expression of the homologous *ftn-2* gene. That *ftn-1(0)* does not reduce resistance to *t*-BOOH also suggests that the cause of this reduction in lifespan may not be increased iron levels.

*ftn-1(0)* does not affect resistance to *t*-BOOH in *daf-2*(+) worms, but it markedly reduces it in *daf-2(m577)* worms. This implies that the high level of overexpression of *ftn-1* in *daf-2* mutants contributes significantly to their resistance to oxidative stress.

The enhancement of *daf-2(m577)* Daf-c and age by *ftn-1(0)* suggests that complete loss of *ftn-1* impacts the signaling pathways that control both dauer larva formation and ageing. This effect of *ftn-1* may affect either insulin/IGF-1 signaling (IIS) itself, one some other pathway upstream or in parallel to IIS. This could either involve an effect on redox status, as influenced by free iron levels, or some other ferritin-mediated process (*e.g.* affecting function of signaling-associated ferroproteins).

The enhancement of *daf-2* Age by *ftn-1(0)* is suppressed by *ftn-2(0)*. This is consistent with functional redundancy between *ftn-1* and *ftn-2*, and may reflect a negative effect on viability of complete absence of ferritin, as seen in *daf-2(+)* worms. The combination of *ftn-1(0)* and *ftn-2(0)* does not reduce *daf-2* Age.

### 4.4 EPR as a tool for measuring *in vivo* ‘free’ iron in *C. elegans*

In this study, we used cw-EPR spectroscopy as a tool to measure levels of ‘free’ iron *in vivo* in *C. elegans*. This proved an effective means to detect increases in free iron levels resulting from iron supplementation. However, in a number of interventions where more subtle changes in iron levels were expected, no changes in free iron levels were detectable by EPR (data not shown). These interventions included iron chelation, *ftn-1* over-expression and *daf-2(m577)*. This suggests that, despite using the most sensitive commercial X-Band spectrometer available, EPR as applied here may be relatively insensitive as a tool for measuring changes in free iron levels close to the normal physiological range. Potentially, a way round this limitation would be to measure free iron content in an even larger numbers of worms or perform the EPR at higher magnetic fields/frequencies.

## 5 Conclusions

In this study we have used an animal model (*C. elegans*) to test the idea that ROS production catalyzed by free iron contributes to organismal ageing. We have used several approaches to manipulate free iron levels, and find that in a number of cases these can influence peroxide resistance and damage levels without affecting lifespan. Severe loss of *ftn* gene function can reduce lifespan in some contexts, but it remains unclear whether this is a function of increased oxidative stress. The results presented here demonstrate the importance of ferritins in organismal resistance to oxidative stress, which may reflect effects of ferritins on free iron levels. However, they also suggest free iron levels are not a determinant of ageing in *C. elegans* under standard culture conditions. This is consistent with a number of other studies in recent years that imply that oxidative damage (and perhaps molecular damage) do not cause ageing in this organism.

## Contributors

D. Gems conceived of the study and designed most of the experiments. S. Valentini, F. Cabreiro, D. Ackerman, M. Alam, M. Kunze carried out the experimental work. C.W.M. Kay contributed to design of, and oversaw, the EPR experiments. S. Valentini and D. Gems wrote the manuscript. D. Gems supervised the project. All authors read and approved the manuscript.

## Footnotes

Appendix A Supplementary data associated with this article can be found, in the online version, at doi:10.1016/j.mad.2012.03.003.

## References

[bib0005] Ackerman D., Gems D. (2012). Insulin/IGF-1 and hypoxia signaling act in concert to regulate iron homeostasis in /*Caenorhabditis elegans*/. PLoS Genet.

[bib0010] Crichton R.R., Ward R.J. (1992). Iron metabolism – new perspectives in view. Biochemistry.

[bib0015] Doonan R., McElwee J.J., Matthijssens F., Walker G.A., Houthoofd K., Back P., Matscheski A., Vanfleteren J.R., Gems D. (2008). Against the oxidative damage theory of aging: superoxide dismutases protect against oxidative stress but have little or no effect on life span in *Caenorhabditis elegans*. Genes and Development.

[bib0020] Evans, T.C., 2006. Transformation and microinjection, In The *C. elegans* research community, Wormbook.

[bib0025] Fridovich I. (1978). The biology of oxygen radicals. Science.

[bib0030] Galaris D., Mantzaris M., Amorgianiotis C. (2008). Oxidative stress and aging: the potential role of iron. Hormones (Athens).

[bib0035] Gems D., Doonan R. (2009). Antioxidant defense and aging in *C. elegans*: is the oxidative damage theory of aging wrong?. Cell Cycle.

[bib0040] Gourley B.L., Parker S.B., Jones B.J., Zumbrennen K.B., Leibold E.A. (2003). Cytosolic aconitase and ferritin are regulated by iron in *Caenorhabditis elegans*. Journal of Biological Chemistry.

[bib0045] Gutteridge J.M., Halliwell B. (2000). Free radicals and antioxidants in the year 2000. A historical look to the future. Annals of New York Academy of Science.

[bib0050] Halliwell B., Gutteridge J.M. (1984). Oxygen toxicity, oxygen radicals, transition metals and disease. Biochemical Journal.

[bib0055] Harman D. (1956). Aging: a theory based on free radical and radiation chemistry. Journal of Gerontology.

[bib0060] Heidler T., Hartwig K., Daniel H., Wenzel U. (2010). *Caenorhabditis elegans* lifespan extension caused by treatment with an orally active ROS-generator is dependent on DAF-16 and SIR-2.1. Biogerontology.

[bib0065] Honda Y., Honda S. (1999). The daf-2 gene network for longevity regulates oxidative stress resistance and Mn-superoxide dismutase gene expression in *Caenorhabditis elegans*. Federation of American Socety for Experimental Biology Journal.

[bib0070] Hu P.J.D. (2007). Dauer.

[bib0075] Imlay J.A., Chin S.M., Linn S. (1988). Toxic DNA damage by hydrogen peroxide through the Fenton reaction in vivo and in vitro. Science.

[bib0080] Kamath R., Fraser A., Dong Y., Poulin G., Durbin R., Gotta M., Kanapin A., Le Bot N., Moreno S., Sohrmann M. (2003). Systematic functional analysis of the *Caenorhabditis elegans* genome using RNAi. Nature.

[bib0085] Kamath R.S., Ahringer J. (2003). Genome-wide RNAi screening in *Caenorhabditis elegans*. Methods.

[bib0090] Kamath R.S., Martinez-Campos M., Zipperlen P., Fraser A.G., Ahringer J. (2001). Effectiveness of specific RNA-mediated interference through ingested double-stranded RNA in *Caenorhabditis elegans*. Genome Biology.

[bib0095] Keaney M., Gems D. (2003). No increase in lifespan in *Caenorhabditis elegans* upon treatment with the superoxide dismutase mimetic EUK-8. Free Radical Biology and Medicine.

[bib0100] Keaney M., Matthijssens F., Sharpe M., Vanfleteren J.R., Gems D. (2004). Superoxide dismutase mimetics elevate superoxide dismutase activity *in vivo* but do not retard aging in the nematode *Caenorhabditis elegans*. Free Radical Biology and Medicine.

[bib0105] Kenyon C., Chang J., Gensch E., Rudener A., Tabtiang R. (1993). A *C. elegans* mutant that lives twice as long as wild type. Nature.

[bib0110] Keyer K., Gort A.S., Imlay J.A. (1995). Superoxide and the production of oxidative DNA damage. Journal of Bacteriology.

[bib0115] Keyer K., Imlay J.A. (1996). Superoxide accelerates DNA damage by elevating free-iron levels. Proceedings of National Academy of Sciences of the United States of America.

[bib0120] Kim J., Takahashi M., Shimizu T., Shirasawa T., Kajita M., Kanayama A., Miyamoto Y. (2008). Effects of a potent antioxidant, platinum nanoparticle, on the lifespan of *Caenorhabditis elegans*. Mechanisms of Ageing and Development.

[bib0125] Kim Y.I., Cho J.H., Yoo O.J., Ahnn J. (2004). Transcriptional regulation and life-span modulation of cytosolic aconitase and ferritin genes in *C. elegans*. Journal of Molecular Biology.

[bib0130] Kimura K.D., Tissenbaum H.A., Liu Y., Ruvkun G. (1997). daf-2, an insulin receptor-like gene that regulates longevity and diapause in *Caenorhabditis elegans*. Science.

[bib0135] Larsen P.L. (1993). Aging and resistance to oxidative stress in *Caenorhabditis elegans*. Proceedings of National Academy of Sciences of the United States of America.

[bib0140] Levi S., Arosio P. (2004). Mitochondrial ferritin. International Journal of Biochemistry and Cell Biology.

[bib0145] Liochev S.I. (1999). The mechanism of Fenton-like reactions and their importance for biological systems. A biologist's view. Metal Ions in Biological Systems.

[bib0150] Lithgow G., Walker G. (2002). Stress resistance as a determinate of *C. elegans* lifespan. Mechanisms of Ageing and Development.

[bib0155] Lithgow G.J., White T.M., Melov S., Johnson T.E. (1995). Thermotolerance and extended life-span conferred by single-gene mutations and induced by thermal stress. Proceedings of National Academy of Sciences of the United States of America.

[bib0160] McElwee J.J., Schuster E., Blanc E., Piper M.D., Thomas J.H., Patel D.S., Selman C., Withers D.J., Thornton J.M., Partridge L., Gems D. (2007). Evolutionary conservation of regulated longevity assurance mechanisms. Genome Biology.

[bib0165] McElwee J.J., Schuster E., Blanc E., Thomas J.H., Gems D. (2004). Shared transcriptional signature in *C. elegans* dauer larvae and long-lived *daf-2* mutants implicates detoxification system in longevity assurance. Journal of Biology Chemistry.

[bib0170] Medvedev Z.A. (1990). An attempt at a rational classification of theories of ageing. Biological Reviews.

[bib0175] Melov S., Ravenscroft J., Malik S., Gill M., Walker D., Clayton P., Wallace D., Malfroy B., Doctrow S., Lithgow G. (2000). Extension of life-span with superoxide dismutase/catalase mimetics. Science.

[bib0180] Meneghini R. (1997). Iron homeostasis, oxidative stress, and DNA damage. Free Radical Biology and Medicine.

[bib0185] Miyabayashi T., Palfreyman M.T., Sluder A.E., Slack F., Sengupta P. (1999). Expression and function of members of a divergent nuclear receptor family in *Caenorhabditis elegans*. Developmental Biology.

[bib0190] Muller F.L., Lustgarten M.S., Jang Y., Richardson A., Van Remmen H. (2007). Trends in oxidative aging theories. Free Radical Biology and Medicine.

[bib0195] Murakami S., Johnson T.E. (1996). A genetic pathway conferring life extension and resistance to UV stress in *Caenorhabditis elegans*. Genetics.

[bib0200] Mwebi N.O. (2005). Fenton & Fenton-Like Reactions: The Nature Of Oxidizing Intermediates Involved.

[bib0205] Pate K.T., Rangel N.A., Fraser B., Clement M.H., Srinivasan C. (2006). Measuring free iron levels in *Caenorhabditis elegans* using low-temperature Fe(III) electron paramagnetic resonance spectroscopy. Analytical Biochemistry.

[bib0210] Perez V.I., Bokov A., Van Remmen H., Mele J., Ran Q., Ikeno Y., Richardson A. (2009). Is the oxidative stress theory of aging dead?. Biochimica et Biophysica Acta.

[bib0215] Polla A.S., Polla L.L., Polla B.S. (2003). Iron as the malignant spirit in successful ageing. Ageing Research Reviews.

[bib0220] Polla B.S. (1999). Therapy by taking away: the case of iron. Biochemical Pharmacology.

[bib0225] Puntarulo S., Cederbaum A.I. (1996). Role of cytochrome P-450 in the stimulation of microsomal production of reactive oxygen species by ferritin. Biochimca et Biophysica Acta.

[bib0230] Schulz T.J., Zarse K., Voigt A., Urban N., Birringer M., Ristow M. (2007). Glucose restriction extends *Caenorhabditis elegans* life span by inducing mitochondrial respiration and increasing oxidative stress. Cell Metabolism.

[bib0235] Sohal R.S., Weindruch R. (1996). Oxidative stress, caloric restriction, and aging. Science.

[bib0240] Srinivasan C., Liba A., Imlay J.A., Valentine J.S., Gralla E.B. (2000). Yeast lacking superoxide dismutase(s) show elevated levels of free iron as measured by whole cell electron paramagnetic resonance. Journal of Biological Chemistry.

[bib0245] Terman A., Gustafsson B., Brunk U.T. (2006). The lysosomal-mitochondrial axis theory of postmitotic aging and cell death. Chemico-Biological Interactions.

[bib0250] Tullet J.M., Hertweck M., An J.H., Baker J., Hwang J.Y., Liu S., Oliveira R.P., Baumeister R., Blackwell T.K. (2008). Direct inhibition of the longevity-promoting factor SKN-1 by insulin-like signaling in *C. elegans*. Cell.

[bib0255] Uchiyama S., Koike H., Shimizu T., Shirasawa T. (2005). A superoxide dismutase/catalase mimetic extends the lifespan of short-lived mev-1 mutant but not the wild type strain *Caenorhabditis elegans*. Anti-ageing Medical Research.

[bib0260] Van Raamsdonk J.M., Hekimi S. (2009). Deletion of the mitochondrial superoxide dismutase *sod-2* extends lifespan in *Caenorhabditis elegans*. PLoS Genetics.

[bib0265] Van Raamsdonk J.M., Hekimi S. (2010). Reactive oxygen species and aging in *Caenorhabditis elegans*: Causal or casual relationship?. Antioxidants and Redox Signaling.

[bib0270] Vanfleteren J.R. (1993). Oxidative stress and ageing in *Caenorhabditis elegans*. Biochemical Journal.

[bib0275] Vile G.F., Tyrrell R.M. (1993). Oxidative stress resulting from ultraviolet A irradiation of human skin fibroblasts leads to a heme oxygenase-dependent increase in ferritin. Journal of Biological Chemistry.

[bib0280] Wang J., Kim S. (2003). Global analysis of dauer gene expression in *Caenorhabditis elegans*. Development.

[bib0285] Yang W., Hekimi S. (2010). A mitochondrial superoxide signal triggers increased longevity in *Caenorhabditis elegans*. PLoS Biology.

[bib0290] Yang W., Li J., Hekimi S. (2007). A Measurable increase in oxidative damage due to reduction in superoxide detoxification fails to shorten the life span of long-lived mitochondrial mutants of *Caenorhabditis elegans*. Genetics.

