# Supplementary material for: Manipulation of in vivo iron levels can alter resistance to oxidative stress without affecting ageing in the nematode C. elegans
Source: Mech Ageing Dev. 2012 May;133(5):282–90. doi: 10.1016/j.mad.2012.03.003 (PMC3449239; doi:10.1016/j.mad.2012.03.003)
Supplement: Supplementary file 1 [file mmc1.doc]

**Supplementary data**

**Figures S1-3**

**Tables S1-S14**


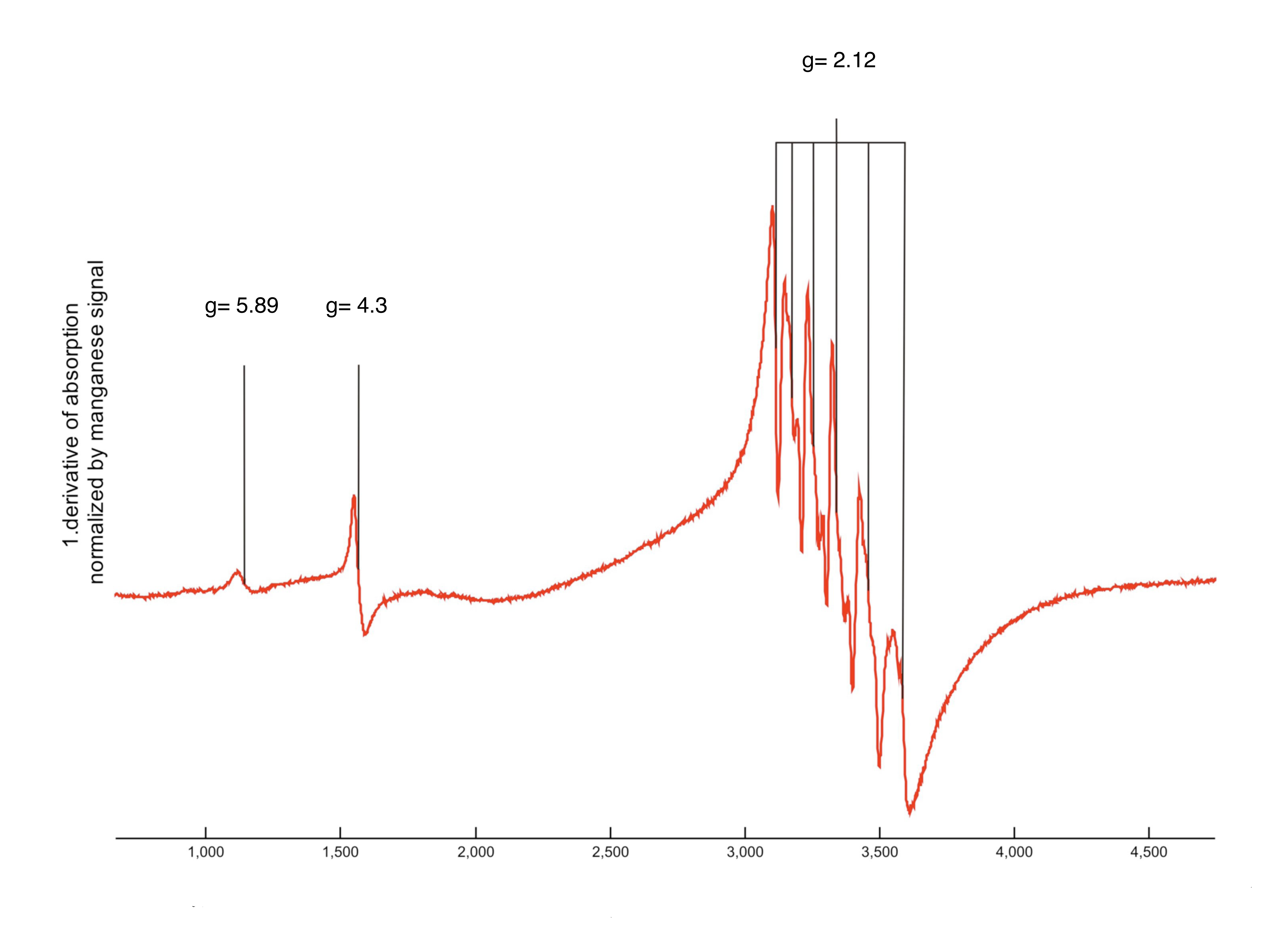


Figure S1: EPR spectra of wildtype worms. The EPR spectra detected a heme signal at *g*=5.89, an iron (III) signal at *g*=4.3 and a manganese signal at *g*=2.12. The manganese signal was used to standardize all the samples.

Figure S2: Effect of iron supplementation in the worm. Genotype: N2 wildtype. (A) EPR measurement represent the Spectrum. It was not possible to do statistical analysis with wildtype animals on 5 mM and 50 mM iron plates, since the measurement at these concentrations was only performed once. (C, D) Protein carbonylation is increased by 15 mM iron supplementation. (C) Statistical analysis of four biological replicates showing carbonylated protein levels normalized to actin immunoreactivity. (D) Representative oxyblot showing the carbonylated protein and actin levels. Error bars, S.E.M. (E) 15 mM iron increases peroxide (*t*-BOOH) toxicity and reduces lifespan and (F).

Figure S3: Effect of *ftn-1* mutation on *C. elegans* lifespan and oxidative stress resistance. The trials were performed at 25˚C. Genotype: N2 wildtype, *daf-2*(-) DR1567 *daf-2(m577) III*, *ftn-1*(-) GA912 *ftn-1(ok3625) V* and *daf-2*(-) *ftn-1*(-) *daf-2(m577) III; ftn-1(ok3625) V*. (A, C, D) Survival of *ftn-1* mutants in wildtype and *daf-2* mutant background. (B, D, F) Peroxide resistance of *ftn-1* mutants in wildtype and *daf-2* mutant background (*tert*-butyl hydroperoxide).

Table S1: Effect of iron supplementation on free iron levels in the worm. Genotype: N2 wildtype. The numbers for each EPR measurement represent the length of the iron peak. Statistical analysis was done with the Student’s T-test. It was not possible to do statistical analysis with wildtype animals on 5 mM and 50 mM iron plates, since the measurement at these concentrations was only performed once. FAC, ferric ammonium citrate. [n] Independent biological replicate number.

| **Strain** | **FAC (mM)** | **Size of iron peak (arbitrary units)** | **% of control** | ***p* *vs.* control** |
| --- | --- | --- | --- | --- |
| Wildtype | Ctrl | **[C] 0.37**  [1] 0.50  [2] 0.37  [3] 0.24  **[C_2_] 0.0241**  [4] 0.0162  [5] 0.0266  [6] 0.0296 |  |  |
| Wildtype | 5 | [3] 0.50 | +108.33 |  |
| Wildtype | 9 | **[C_2_] 0.0706**  [4] 0.0494  [5] 0.0905  [6] 0.0706 | **+192.81**  +204.93  +240.22  +143.58 | **0.03** |
| Wildtype | 15 | **[C] 1.60**  [1] 1.41  [2] 1.76  [3] 1.63 | **+332.43**  +182  +375.67  +579.94 | **0.0004** |
| Wildtype | 50 | [3] 2.39 | +895.83 |  |

Table S2: Effect of iron supplementation on peroxide toxicity. The trials were performed at room temperature on 10 mM *t*-BOOH plates. Genotype: N2 wildtype, *p*, log rank test. [n] biological replicates number.

| **Strain** | **+10 mM *t*-BOOH** | **Deaths/ censored** | **Mean life span (hours)** | **% of control** | ***p* *vs.* control** |
| --- | --- | --- | --- | --- | --- |
| Wildtype | Control | **[C] 201/10**  [1] 25/0  [2] 29/0  [3] 43/4  [4] 58/2  [5] 46/4 | **6.87**  6.2  5.58  7.67  6.75  7.40 |  |  |
| Wildtype | 5 | **[C] 62/4**  [1] 25/0  [3] 37/4 | **6.61**  5.92  7.08 | **-3.78**  -4.51  -7.69 | **0.32**  0.27  0.21 |
| Wildtype | 8 | **[C] 100/3**  [4] 58/3  [5] 42/0 | **6.94**  6.65  7.33 | **+1.01**  -1.48  -0.94 | **0.85**  0.88  0.35 |
| Wildtype | 15 | **[C] 95/7**  [2] 42/0  [3] 53/7 | **5.74**  4.23  6.94 | **-16.44**  -24.19  -9.69 | **<.0001**  <.0001  0.06 |
| Wildtype | 25 | [1] 23/0 | 5.47 | -11.77 | 0.06 |

Table S3: Effect of iron supplementation on lifespan. The trials were performed at 20˚C without FUdR, except for trial [4] where 10 µM FUdR was used. Genotype: N2 wildtype, *p*, log rank test. [n] biological replicates number.

| **Strain** | **FAC (mM)** | **Deaths/ censored** | **Mean life span (days)** | **% of control** | ***p* *vs.* control** |
| --- | --- | --- | --- | --- | --- |
| Wildtype | Control | **[C] 214/132**  [1] 30/20  [2] 97/28  [3] 21/20  [4] 66/64  [5] 100/34 | **15.93**  15.70  14.80  18.29  16.32  17.49 |  |  |
| Wildtype | 5 | [1] 28/22  [5] 124/23 | 16.24  19.09 | +3.43  +9.14 | 0.47  0.05 |
| Wildtype | 8 | [4] 88/44 | 16.70 | +2.32 | 0.42 |
| Wildtype | 10 | **[C] 130/48**  [1] 30/20  [4] 100/28 | **15.59**  13.16  16.28 | **-2.13**  -16.17  -0.24 | **0.98**  0.01  0.96 |
| Wildtype | 15 | [1] 29/21  [5] 63/69 | 11.35  15.92 | -27.70  -8.97 | <.0001  0.06 |
| Wildtype | 25 | **[C] 153/12**  [2] 123/2  [3] 30/10 | **12.24**  11.54  14.88 | **-23.16**  -22.02  -18.64 | **<.0001**  <.0001  <.0001 |
| Wildtype | 50 | [3] 36/6 | 14.41 | -21.21 | <.0001 |

Table S4: Effect of 9 mM iron supplementation on peroxide toxicity. The trials were performed at room temperature on 10 mM *t*-BOOH plates. Genotype: N2 wildtype, *p*, log rank test. [n] biological replicates number.

| **Strain** | **+ 10 mM *t*-BOOH** | **Deaths/ censored** | **Mean life span (hours)** | **% of control** | ***p* *vs.* control** |
| --- | --- | --- | --- | --- | --- |
| Wildtype | Control | **[C] 165/6**  [1] 58/2  [2] 46/4  [3] 61/0 | **7.69**  6.75  7.40  8.81 |  |  |
| Wildtype | 9 | **[C] 138/19**  [1] 51/9  [2] 32/9  [3] 55/1 | **6.85**  6.19  6.31  7.78 | **-10.92**  -8.29  -14.72  -11.69 | **<.0001**  0.01  0.007  0.0001 |

Table S5: Effect of 9 mM iron supplementation on lifespan. The *t*-BOOH trials were performed at room temperature on 10 mM *t*-BOOH plates. The lifespan trials were performed at 20˚C without FUdR, except of trial [4] 10 µM FUdR was used. Genotype: N2 wildtype, *p*, log rank test. [n] biological replicates number.

| **Strain** | **FAC (mM)** | **Deaths/ censored** | **Mean life span (days)** | **% of control** | ***p* *vs.* control** |
| --- | --- | --- | --- | --- | --- |
| Wildtype | Control | **[C] 306/122**  [1] 66/64  [2] 115/21  [3] 125/37 | **15.05**  16.32  14.24  14.87 |  |  |
| Wildtype | 9 | **[C] 287/103**  [1] 95/40  [2] 83/37  [3] 109/30 | **15.13**  16.16  13.90  15.21 | **0.53**  -0.98  -2.38  +2.01 | **0.77**  0.81  0.43  0.65 |

Table S6: Effect of 100 µM deferoxamine on peroxide toxicity. The *t*-BOOH trials were performed at room temperature on 10 mM *t*-BOOH plates. Genotypes: N2 wildtype, *p*, log rank test. [n] biological replicates number.

| **Strain** | **+10 mM *t*-BOOH** | **Deaths/ censored** | **Mean life span (days)** | **% of control** | ***p* *vs.* control** |
| --- | --- | --- | --- | --- | --- |
| Wildtype | Control | **[C] 97/4**  [1] 25/0  [2] 29/0  [3] 43/4 | **6.67**  6.2  5.58  7.67 |  |  |
| Wildtype | 100 µM DF | **[C] 120/8**  [1] 26/0  [2] 50/3  [3] 44/5 | **7.61**  9.46  5.01  9.34 | **+14.09**  +52.58  -10.21  +21.77 | **0.008**  <.0001  0.08  0.005 |

Table S7: Effect of *ftn-1* over-expression (OE) on peroxide toxicity in *C. elegans*. The trials were performed at room temperature on 10 mM *t*-BOOH plates. Genotype: N2 wildtype, *ftn-1* OE GA904 (*wuEx187 [Pftn-1::ftn-1::ftn-13’UTR + coel::GFP]*) and the injection marker control *ftn-1* OE ctrl GA901 (*wuEx188 [coel::GFP]*). *p*, log rank test. [n] biological replicate number.

| **Strain** | **Deaths/ censored** | **Mean life span (hours)** | **% *vs.* wildtype** | ***p* *vs.* wildtype** | **% *vs.* *ftn-1* OE control** | ***p* *vs.* *ftn-1* OE control** |
| --- | --- | --- | --- | --- | --- | --- |
| Wildtype | **[C] 320/11**  [1] 49/0  [2] 25/0  [3] 45/7  [4] 29/0  [5] 43/4  **[C_1_] 129/0**  [6] 68/0  [7] 61/0 | **6.95**  4.85  6.2  5.93  5.58  7.67  **8.32**  7.88  8.81 |  |  |  |  |
| *ftn-1* OE ctrl | **[C_1_] 111/5**  [6] 57/0  [7] 54/5 | **7.85**  7.57  8.14 | **-5.64**  -3.93  -7.60 | **0.11**  0.62  0.02 |  |  |
| *ftn-1* OE | **[C] 299/16**  [1] 48/0  [2] 25/0  [3] 40/10  [4] 36/0  [5] 30/6  **[C_1_] 120/0**  [6] 60/0  [7] 60/0 | **7.69**  6.27  6.6  7.2  7.33  7.81  **8.73**  9  8.46 | **+10.64**  +29.27  +6.45  +21.41  +31.36  +1.82  **+4.92**  +14.21  -3.97 | **0.0002**  0.0001  0.35  0.0002  0.002  0.96  **0.06**  0.006  0.19 | **+11.21**  +18.89  +3.93 | **0.005**  0.005  0.43 |

Table S8: Effect of 100 µM deferoxamine on lifespan. The trials were performed at 25˚C. Genotypes: SS104 *glp-4(bn2),* N2 wildtype, *p*, log rank test. [n] biological replicate number.

| **Strain** | **Treatment** | **Deaths/ censored** | **Mean life span (days)** | **% of control** | ***p* *vs.* control** |
| --- | --- | --- | --- | --- | --- |
| SS104 | Control | **[C] 133/22**  [1] 22/8  [2] 111/14 | **13.93**  12.54  14.20 |  |  |
| SS104 | 100 μM DF | **[C] 144/11**  [1] 27/3  [2] 117/8 | **13.31**  11.62  13.68 | **-4.45**  -7.33  -3.66 | **0.07**  0.23  0.09 |

Table S9: Effect of *ftn-1* OE on life span in *C. elegans*. The trials were performed at 20°C without FUdR. Genotype: N2 wildtype, *ftn-1* OE GA904 (*wuEx187 [Pftn-1::ftn-1::ftn-13’UTR + coel::GFP]*) and the injection marker control *ftn-1* OE control GA901 (*wuEx188 [coel::GFP]*). *p*, log rank test. [n] biological replicates number.

| **Strain** | **Deaths/ censored** | **Mean life span (days)** | **% *vs.* wildtype** | ***p* *vs.* wildtype** | **% *vs.* *ftn-1* OE control** | ***p* *vs.* *ftn-1* OE control** |
| --- | --- | --- | --- | --- | --- | --- |
| Wildtype | **[C] 306/149**  [1] 21/20  [2] 94/33  [3] 66/59  [4] 125/37 | **17.54**  18.29  19.63  18.89  14.87 |  |  |  |  |
| *ftn-1* OE ctrl | **[C] 270/118**  [2] 66/55  [3] 80/34  [4] 124/29 | **17.67**  20.22  18.04  15.72 | **+0.74**  +3  -4.49  +5.71 | **0.64**  0.26  0.34  0.05 |  |  |
| *ftn-1* OE | **[C] 266/164**  [1] 20/14  [2] 58/62  [3] 67/58  [4] 121/30 | **17.98**  19.83  21.39  18.25  15.63 | **+2.50**  +8.41  +8.96  -3.38  +5.11 | **0.29**  0.11  0.02  0.36  0.08 | **+1.75**  +5.78  +1.16  -0.57 | **0.58**  0.19  0.97  0.78 |

Table S10: Effect of *ftn-1* RNAi on life span in *daf-2* mutants. The trials were performed at 25˚C, without FUdR. Ctrl RNAi is the L4440 plasmid vector. [C] combined data. Genotypes of *daf-2*(+) and *daf-2*: NL2099 (*rrf-3(pk1426) II),* GA303 (*rrf-3(pk1426) II; daf-2(m577) III)* respectively*, p*, probability of being the same as specified control (log rank). [n] Independent biological replicate number.

| - **Strain** | - **RNAi** | - **Deaths/ censored** | - **Mean life span (days)** | - **% *vs.*** - ***daf-2*(+) ctrl RNAi** | - ***p* *vs.*** - ***daf-2*(+) ctrl RNAi** | - **% *vs.* *daf-2* ctrl RNAi** | - ***p* *vs.* *daf-2* ctrl RNAi** |
| --- | --- | --- | --- | --- | --- | --- | --- |
| - *daf-2*(+) | - Control | - **[C] 232/18** - [1] 112/13 - [2] 120/5 | - **15.55** - 14.53 - 16.48 |  |  |  |  |
| - *daf-2*(+) | - *ftn-1* | - **[C] 233/17** - [1] 116/9 - [2] 117/8 | - **15.46** - 15.54 - 15.40 | - **-0.57** - +6.95 - -6.55 | - **0.98** - 0.19 - 0.07 |  |  |
| - *daf-2*(+) | - *daf-16* | - **[C] 172/78** - [1] 80/45 - [2] 92/33 | - **12.83** - 12.73 - 12.90 | - **-17.49** - -12.38 - -21.72 | - **<.0001** - 0.0007 - <.0001 |  |  |
| - *daf-2*(+) | *ftn-1/ftn-2* | - [2] 119/7 | - 15.72 | - -4.61 | - 0.12 |  |  |
| - *daf-2* | - Control | - **[C] 233/17** - [1] 114/11 - [2] 119/6 | - **31.47** - 29.85 - 33.02 | - **+102.37** - +105.43 - +100.36 | - **<.0001** - <.0001 - <.0001 |  |  |
| - *daf-2* | - *ftn-1* | - **[C] 241/13** - [1] 121/4 - [2] 120/5 | - **31.35** - 30.68 - 32.02 | - **+101.60** - +111.14 - +94.29 | - **<.0001** - <.0001 - <.0001 | - **-0.38** - +2.78 - -3.02 | - **0.72** - 0.79 - 0.53 |
| - *daf-2* | - *daf-16* | - **[C] 226/25** - [1] 114/12 - [2] 112/13 | - **15.34** - 16.95 - 13.71 | - **-1.35** - +16.65 - -16.80 | - **0.06** - 0.10 - <.0001 | - **-51.25** - -43.21 - -58.48 | - **<.0001** - <.0001 - <.0001 |
| - *daf-2* | *ftn-1/ftn-2* | - [2] 127/5 | - 31.72 | - +92.48 | - <.0001 | - -3.93 | - 0.26 |

Table S11: Effect of *ftn-1* RNAi on peroxide toxicity in *daf-2* mutants. The *t*-BOOH trials were performed at room temperature on 10 mM *t*-BOOH plates. Genotypes of *daf-2*(+) and *daf-2*: NL2099 (*rrf-3(pk1426) II),* GA303 (*rrf-3(pk1426) II; daf-2(m577) III)* respectively*, p*, probability of being the same as specified control (log rank). [n] Independent biological replicate number.

| **Strain** | **RNAi** | **Deaths/ censored** | **Mean life span (hours)** | **% *vs.* genetic control** | ***p* *vs.* genetic control** | **% *vs.***  ***daf-2*(+) control** | ***p* *vs.***  ***daf-2*(+) control** |
| --- | --- | --- | --- | --- | --- | --- | --- |
| *daf-2* (+) | Control | 52/5 | 6.28 |  |  |  |  |
| *daf-2* (+) | *ftn-1* | 50/11 | 5.58 | -11.14 | 0.01 |  |  |
| *daf-2* | Control | 46/12 | 10.5 |  |  | +67.19 | <.0001 |
| *daf-2* | *ftn-1* | 51/10 | 8.54 | -18.33 | 0.06 | +35.98 | 0.002 |

Table S12: Effect of *ftn-1* mutation on peroxide toxicity in *C. elegans*. The trials were performed at 25C on 10 mM *t*-BOOH plates. Genotype: N2 wildtype, *daf-2*(-) DR1567 *daf-2(m577) III*, *ftn-1*(-) GA912 *ftn-1(ok3625) V* and *daf-2*(-) *ftn-1*(-) GA931 *daf-2(m577) III, ftn-1(ok3625) V*. *p*, log rank test. [n] biological replicate number.

| **Genotype** | **Deaths/ censored** | **Mean life span (hours)** | **% *vs.* wildtype** | ***p* *vs.* wildtype** | **% *vs.* genetic ctrl** | ***p* *vs.* genetic ctrl** |
| --- | --- | --- | --- | --- | --- | --- |
| Wildtype | **[C] 252/23**  [1] 46/0  [2] 51/9  [3] 51/6  [4] 55/3  [5] 49/5 | **6.61**  6.13  7.09  8.23  6.03  5.55 |  |  |  |  |
| *daf-2* (-) | **[C] 226/43**  [1] 37/14  [2] 52/9  [3] 50/13  [4] 46/3  [5] 41/4 | **9.51**  9.74  9.23  10.36  10.26  7.80 | **+43.87**  +58.89  +30.18  +25.88  +70.14  +40.54 | **<.0001**  <.0001  <.0001  0.0001  <.0001  <.0001 |  |  |
| *ftn-1* (-) | **[C] 256/28**  [1] 35/10  [2] 62/4  [3] 61/5  [4] 48/5  [5] 50/4 | **6.76**  6.45  6.93  8.72  6  5.14 | **+2.26**  +5.22  -2.25  +5.95  -0.49  -7.38 | **0.47**  0.30  0.44  0.42  0.61  0.24 |  |  |
| *daf-2* (-)*, ftn-1*(-) | **[C] 257/37**  [1] 51/12  [2] 55/5  [3] 56/8  [4] 46/8  [5] 49/4 | **8.37**  10.78  8.72  8.07  8  6.16 | **+26.62**  +75.85  +22.99  -1.94  +32.66  +10.99 | **<.0001**  <.0001  <.0001  0.40  0.0003  0.06 | **-11.98**  +10.67  -5.52  -22.10  -22.02  -21.02 | **0.0004**  0.11  0.84  <.0001  0.009  0.01 |

Table S13: Effect of *ftn-1* mutation on *C. elegans* lifespan. The trials were performed at 25˚C. Genotype: N2 wildtype, *daf-2*(-) DR1567 *daf-2(m577) III*, *ftn-1*(-) GA912 *ftn-1(ok3625) V* and *daf-2*(-) *ftn-1*(-) GA931 *daf-2(m577) III; ftn-1(ok3625) V, daf-2*(-) *ftn-1*(-) *ftn-2 (-)* GA933 *ftn-2(ok404) I; daf-2(m577) III; ftn-1(ok3625) V*. *p*, log rank test. [n] biological replicate number.

| **Strain** | **Deaths/ censored** | **Mean life span (days)** | **% *vs.* wildtype** | ***p* *vs.* wildtype** | **% *vs.* genetic ctrl** | ***p* *vs.* genetic (treatment) ctrl** | **% vs. double to triple mutant** | ***p* vs. double to triple mutant** |
| --- | --- | --- | --- | --- | --- | --- | --- | --- |
| Wildtype | **[C] 397/266**  [1] 70/76  [2] 125/49  [3] 63/99  [4] 139/42 | **16.28**  10.81  17.34  17.15  17.2 |  |  |  |  |  |  |
| *daf-2* (-) | **[C] 419/278**  [1] 177/14  [2] 95/82  [3] 64/68  [4] 83/114 | **24.92**  19.13  28.43  28.42  28.98 | **+53.07**  +76.96  +63.95  +65.71  +68.48 | **<.0001**  <.0001  <.0001  <.0001  <.0001 |  |  |  |  |
| *ftn-1* (-) | **[C] 400/262**  [1] 66/105  [2] 119/39  [3] 91/48  [4] 124/70 | **13.07**  10.70  11.95  13.56  14.88 | **-19.71**  -1.01  -31.08  -20.93  -13.48 | **<.0001**  0.61  <.0001  <.0001  0.001 |  |  |  |  |
| *ftn-1* (-) *ftn-2* (-) | **[C] 310/303**  [1] 39/132  [2] 107/47  [3] 56/57  [4] 108/67 | **10.96**  10.31  10.93  10.98  11.03 | **-32.67**  -4.62  -36.96  -35.97  -35.87 | **<.0001**  0.98  <.0001  <.0001  <.0001 |  |  |  |  |
| *daf-2* (-)*, ftn-1*(-) | **[C] 581/178**  [1] 165/51  [2] 165/23  [3] 111/54  [4] 140/50 | **27.57**  21.80  31.95  30  26.95 | **+69.34**  +101.66  +84.25  +77.08  +56.68 | **<.0001**  <.0001  <.0001  <.0001  <.0001 | **+10.63**  +13.95  +12.38  +6.86  -7 | **<.0001**  <.0001  0.12  0.38  0.44 |  |  |
| *daf-2* (-)*, ftn-1*(-) *ftn-2* (-) | **[C] 259/442**  [1] 32/144  [2] 91/90  [3] 62/87  [4] 74/121 | **23.27**  19.90  21.97  26.27  23.62 | **+42.93**  +84.08  +26.70  53.17  +37.32 | **<.0001**  <.0001  <.0001  <.0001  <.0001 | **-6.62**  +4.02  -22.72  -7.56  -18.49 | **0.61**  0.16  0.0008  0.88  0.02 | **-15.59**  -8.71  -31.23  -13.5  -12.35 | **0.007**  0.49  0.001  0.32  0.19 |

Table S14: Dauer formation assay of *daf-2 ftn-1 mutants*. Genotypes: N2 wildtype, *daf-2*(-) DR1567 *daf-2(m577) III*, *ftn-1*(-) GA912 *ftn-1(ok3625) V* and *daf-2*(-) *ftn-1*(-) *daf-2(m577) III; ftn-1(ok3625) V*. The dauer formation assay was performed at 23°C.

| **Strain** | **Dauer/ total number of animals** | **% dauer formation (df)** | **% change df *vs.* *daf-2*** |
| --- | --- | --- | --- |
| Wildtype | [1] 0/135  [2] 0/254  [3] 0/145  [4] 0/70  [5] 0/176 | 0  0  0  0  0 |  |
| *ftn-1 (-)* | [1] 0/85  [2] 0/220  [3] 0/53  [4] 0/47  [5] 0/160 | 0  0  0  0  0 |  |
| *daf-2 (-)* | [1] 1/159  [2] 1/74  [3] 5/72  [4] 2/62  [5] 28/143 | 0.62  1.35  6.94  3.22  19.58 |  |
| *daf-2 (-) ftn-1 (-)* | [1] 38/160  [2] 47/88  [3] 2/178  [4] 7/41  [5] 32/158 | 23.75  53.40  1.12  17.07  20.25 | +3,676.25  +3,852.27  -83.82  +429.26  +3.43 |
